# Pediatric critical care nurses' experience with abdominal compartment syndrome

**DOI:** 10.1186/2110-5820-2-S1-S6

**Published:** 2012-07-05

**Authors:** Jennifer Newcombe, Mudit Mathur, Khaled Bahjri, J Chiaka Ejike

**Affiliations:** 1 School of Nursing, Loma Linda University, Loma Linda, CA, USA; 2 Division of Pediatric Critical Care, Department of Pediatrics, School of Medicine, Loma Linda University, 11175 Campus Street, Suite A1117, Loma Linda, CA 92354, USA; 3 Department of Epidemiology and Biostatistics, School of Public Health, Loma Linda University, Loma Linda, CA, USA

**Keywords:** critical care, nurses, abdominal compartment syndrome, intra-abdominal, pressure.

## Abstract

**Background:**

Abdominal compartment syndrome (ACS) is a syndrome associated with multi-system effects of elevated intra-abdominal pressure (IAP) in critically ill children. It has a 90-100% mortality rate if not recognized and treated promptly. Measuring IAP helps identify patients developing intra-abdominal hypertension (IAH) which allows for timely intervention before progression to ACS. IAP helps identify ACS and guides its medical and surgical management. IAP is often measured by the bedside nurse in the intensive care unit. Pediatric critical care nurses (PCCN) play a key role in managing critically ill patients and recognizing potential causes for clinical deterioration such as ACS therefore should be knowledgeable about this entity.

**Objective:**

The aim of this study was to assess the awareness and current knowledge of ACS among PCCN.

**Methods:**

A ten-item written questionnaire was distributed at a National Critical Care Conference in 2006 and again in 2010. Participants of the conference voluntarily completed and immediately returned the survey. Results from the two questionnaires were compared.

**Results:**

Sixty-two percent of 691 questionnaires were completed. The awareness of ACS improved from 69.3% in 2006 to 87.8% in 2010 (*p *< 0.001) among PCCN. "Years in practice" influenced awareness of ACS. Nurses working for 5-10 and > 10 years were, respectively, 2.34 and 1.89 times more likely to be aware of ACS than those working for < 5 years. Hands-on experience managing a child with ACS by PCCN also improved from 49.1% to 67.9% (*p *< 0.001) but remains low. The number of participants who never measured IAP fell from 27.3% to 19.1% (*p *= 0.101). The most common method being used to measure IAP is the bladder method. Knowledge of the definition of ACS remains poor with only 13.2% associating the definition of ACS with organ dysfunction in 2010 which was even lower than in 2006.

**Conclusions:**

There is increasing awareness of ACS and experience in its management among PCCN. However, few PCCN correctly understand the definition of ACS. Since recognition of IAH and early intervention can reduce morbidity and mortality in critically ill patients, further educational efforts should be directed toward improving the knowledge and recognition of ACS by PCCN.

## Introduction

Abdominal compartment syndrome (ACS) arises as a result of persistently elevated intra-abdominal pressure (IAP), ultimately ending in multi-organ dysfunction [1]. Without appropriate management, this condition carries high morbidity, and mortality [1-5].

The World Society of the Abdominal Compartment Syndrome (WSACS; http://www.wsacs.org) recently developed definitions and diagnostic criteria for intra-abdominal hypertension (IAH) and ACS and outlined standards for IAP measurement in adults. It defined ACS as the presence of sustained IAP of 20 mmHg or greater (with or without an abdominal perfusion pressure (APP) of < 60 mmHg) that is associated with new organ dysfunction or failure. It defined IAH as a sustained or repeated pathological elevation in IAP of 12 mmHg or greater [6,7].

Sustained elevation of IAP has multi-system effects. It decreases perfusion to the intra-abdominal organs by increasing pressure on the vena cava and eventually progresses to increased pressure on the aorta and other arterial vessels [5,8]. Over time, it reduces renal plasma flow and glomerular filtration rates [9,10]. This results in decreased urine output contributing to a positive fluid balance, leading to swelling of intra-abdominal organs. The swollen intra-abdominal organs encourage further elevation of IAP which in turn worsens perfusion and propagates a vicious cycle. Increased IAP impacts many organ systems outside the abdominal cavity as well. The cardiovascular effects of IAP include increases in systemic vascular resistance, pulmonary artery pressure, pulmonary artery wedge pressure, and central venous pressure [2,11]. As a consequence, cardiac output decreases and higher filling pressures are required to maintain cardiac output. Additionally, increased IAP impacts pulmonary function. Diaphragmatic elevation occurs, increasing intra-thoracic pressures and decreasing chest wall compliance which exerts a restrictive effect on the lungs, resulting in atelectasis and hypoxia [2,12,13]. Central nervous system manifestations include increases in intracranial pressure and reduced cerebral perfusion pressure [2,14,15]. If sustained clinically relevant elevation in IAP goes unrecognized and untreated, it can lead to multi-system organ failure and death.

Early detection of IAH is essential to the prevention of ACS and requires close surveillance of IAP in patients at increased risk. IAP measurements are often taken by the bedside nurse, and in some cases, initiation of serial IAP monitoring is prompted by pediatric critical care nurses (PCCN). PCCN play an important role in constant observation and recognition of subtle and dynamic changes in the status of critically ill patients in the ICU. Therefore, a PCCN must have a good understanding of the definitions of IAH and ACS and their clinical significance in order to promptly recognize and appropriately manage these conditions as members of the ICU team.

## Objective

The aim of this study was to assess the awareness and current knowledge of ACS among PCCN.

### Materials and methods

A ten-item written questionnaire was designed ("Appendix"). In a pilot study to assess the validity of the survey instrument, the authors evaluated surveys by 63 healthcare providers (HCP). The questionnaire validity was checked by the correlation between items addressing the same objectives. In addition, factor analysis was employed in order to assess the construct validity of the questionnaire.

The internal consistency of ACS awareness was adequate, with significant (*p *< 0.001) Cronbach's alpha coefficient of 0.86. The results of Cronbach's alpha showed between 89.5% and 100% agreement on different questions addressing similar items. On questions where minimum agreement was expected, the degree of agreement ranged between 9.1% and 16.7%. Based on the pilot survey, the questionnaire was considered to be a valid tool.

The Institutional Review Board at Loma Linda University approved this study and waived the requirement for obtaining consent from survey participants. The questionnaire was administered at two national pediatric critical care nursing conferences in 2006 and re-administered in 2010 at one of those conferences. It was distributed to registered participants attending morning sessions of the conferences. Surveys were completed on a voluntary basis and collected at the end of the sessions. The session curricula did not include discussion on IAH and ACS, enabling an assessment of pre-existing knowledge among the survey respondents. The survey questions were designed to determine awareness of ACS as a syndrome, methods of IAP measurement, and diagnostic criteria being used.

### Statistical methods

Descriptive statistics were applied using percentages and proportions. To determine if proportions were different between 2006 and 2010, chi-square tests for independence were used. When assumptions of chi-square were not met, we used Fisher's exact tests. Logistic regression analysis was conducted to assess the association between nurses' awareness of ACS and type of practice or years in practice. The logistic regression model included awareness of ACS as the dependent variable and either "type of practice" or "years in practice" as categorical independent variables. IBM SPSS Statistics 19^© ^(Copyright SPSS Inc. 1989, 2010 Chicago, IL, USA) was used to analyze the data.

## Results

Completed questionnaires were received from 433 of the 691 distributed (62.6% response rate). The majority of respondents (95.5%) were identified as registered nurses and the remaining 4.5% as other healthcare providers. Data from respondents who were not nurses were excluded from analysis.

### Demographics of respondents

**Figure 1:**
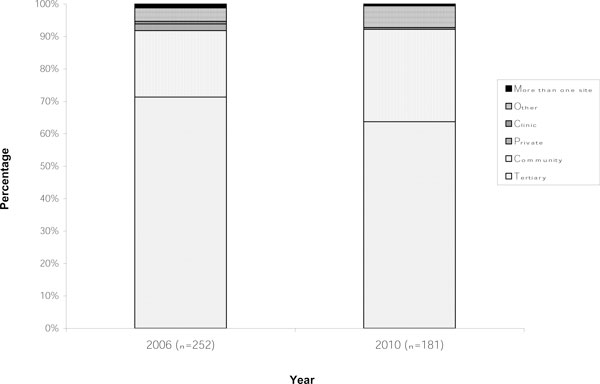
**Demographics: type of practice**.

**Figure 2:**
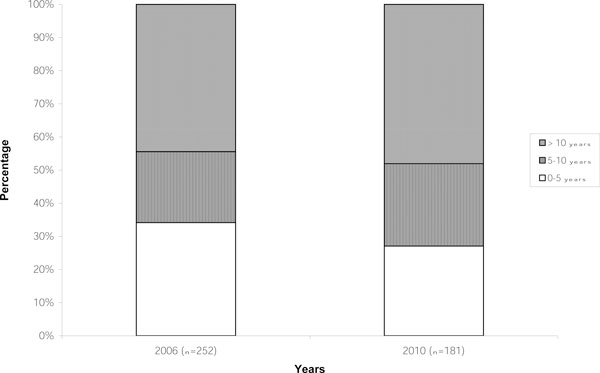
**Demographics: years in practice**.

The response rates for the conferences in 2006 and 2010 were 61.3% and 63.9%, respectively. The majority of participants in this survey worked in the USA (97.2%). Therefore, the findings reflect awareness and current knowledge of PCCN in the USA. Most participants in the survey indicated they worked in tertiary institutions. A small percentage worked in more than one type of institution (Figure 1). Responses to the question regarding years of experience indicated that a large portion of participants had greater than 10 years experience practicing as PCCN (Figure 2). Eighty-five percent of participants in 2006 and 89.5% in 2010 (*p *= 0.201) affirmed they worked in an ICU. There was no statistical difference in the demographics of participants in 2010 compared to 2006.

### Awareness of ACS as an entity

The percentage of PCCN aware of ACS increased from 69.3% in 2006 to 87.8% in 2010, *p *< 0.001 (Table 1). Logistic regression analysis was conducted to assess the association between nurses' awareness of ACS and type of practice or years in practice. The logistic regression model included awareness of ACS as the dependent variable and either "type of practice" or "years in practice" as categorical independent variables. There was no significant association with "types of practice." However, "years in practice" was significantly associated with awareness of ACS. Odds ratio for awareness was 2.34 for those who worked 5-10 years and 1.89 for those who worked greater than 10 years, compared to those who had worked less than 5 years.

**Table 1:** Awareness and experience with abdominal compartment syndrome

	2006	2010	*p *value
Have you heard of ACS?	*n *= 251	*n *= 181	
Yes	69.3%	87.8%	< 0.001^a^

Have you ever managed/cared for a child (0-18 years) with ACS?	*n *= 206	*n *= 179	
Yes	49.1%	67.9%	< 0.001^a^

Do you measure IAP during your management/care of ACS?	*n *= 207	*n *= 168	
No	24.6%	25.0%	NS^a^
Sometimes	44.9%	54.2%	
Yes	30.4%	20.8%	

What method have you used to measure IAP?	*n *= 115	*n *= 135	
Clinical palpation only	22 (19.1%)	9 (6.7%)	0.003^a^
Bladder method only	45 (39.1%)	104 (77.0%)	< 0.001^a^
Direct method only	6 (5.2%)	8 (5.9%)	NS^a^
Esophageal (gastric) only	0 (0.0%)	0 (0.0%)	
Clinical + bladder method	26 (22.6%)	10 (7.4%)	< 0.001^a^
Clinical + direct method	6 (5.2%)	1 (0.7%)	NS^b^

ACS, abdominal compartment syndrome; n, number of subjects; IAP, intra-abdominal pressure. ^a^Chi-square test; ^b^Fisher's exact test

Hands-on experience with management of ACS also improved from 49.1% to 67.9% in the 4-year time span (*p *< 0.001). Fewer nurses indicated that they had not measured IAP during management or care of their patient with ACS, although this decrease did not reach statistical significance. The most common method of measuring IAP remains the bladder method, and the percentage of nurses having direct experience using it almost doubled in 2010 compared to 2006. There is a drop in the sole use of clinical examination to monitor IAP compared to more objective methods. It appears that the intra-esophageal (gastric) method is not being used in pediatric management of ACS.

### Understanding the definition of ACS

The understanding of the definition of ACS among PCCN still appears to be unclear as in 2006, 33.3% defined ACS solely on a value, i.e., how high the IAP reading needs to be and did not relate it to the presence of organ dysfunction. Only 19.5% of respondents correctly defined ACS in children as an elevation in IAP with accompanying multi-organ dysfunction. In 2010, the percentage of PCCN understanding the correct definition of ACS dropped even further to 13.2% (*p *< 0.001) and 52.2% defined it based solely on IAP threshold. The threshold used to define IAP was also variable with the majority indicating the threshold at which ACS would likely develop to be 15-25 mmHg (Table 2).

**Table 2:** Definition of abdominal compartment syndrome

	2006	2010
	
	*n *(%)	*n *(%)
MODS + elevation in IAP	34 (19.5)	21 (13.2)

Specific IAP range without MODS	58 (33.3)	83 (52.2)

Selected threshold		
0-10 mmHg	2 (3.6)	0 (0.0)
10-15 mmHg	16 (28.6)	9 (10.8)
15-25 mmHg	37(66.1)	49 (59.0)
> 25 mmHg	3 (5.4)	25 (30.1)

MODS, multi-organ dysfunction; IAP, intra-abdominal pressure.

## Discussion

In recent years, there has been greater focus on ACS as a significant factor in development of organ failure and mortality in ICU patients. Recognition of IAH, risk factors and clinical signs of ACS, as well as knowledge of management principles can reduce morbidity and mortality in ICU patients. IAH cannot be recognized without measuring IAP because it has no specific clinical presentation. Its definition is based on IAP. The terms IAH and ACS are often inappropriately interchanged and confused by healthcare professionals. There is a critical threshold at which organ dysfunction from elevations in IAP occurs. This threshold varies from one individual to another depending on physiology, co-morbidities, and underlying etiology. It is at that point that IAH becomes ACS. Urgent medical and often surgical interventions need to be applied to alleviate IAP and restore organ perfusion, in order to reverse organ dysfunction caused by ACS. The primary goal in the management of IAH is to decrease the IAP and prevent development of ACS altogether. Each member of the ICU team needs to be aware of IAH and ACS. A clear understanding of the definition and pathophysiology of IAH and ACS is needed, to recognize, distinguish, and diagnose these two entities and subsequently manage them appropriately. Several written surveys to appreciate the awareness, knowledge, and management approaches to ACS among different healthcare providers have been performed [1,16-20]. This is the first written survey on the awareness of ACS directed at PCCN and the first to assess the progression of awareness over time.

In this survey, there was a rise in the awareness of ACS among PCCN between 2006 and 2010. The percentage of PCCN who reported managing a child with ACS themselves also rose, indicating greater hands-on experience with ACS management. A recent survey of pediatric healthcare providers, attending two pediatric critical care conferences, revealed that 22% of participants never heard of ACS and two thirds of those who were aware indicated personal experience with its management. In the same survey, 97% of pediatric intensivists were aware of ACS as an entity and pediatric healthcare providers working in ICUs tended to be more aware of it also [16]. Tiwari et al. surveyed clinical directors of ICUs in the UK and found a greater awareness among directors in teaching hospitals compared to general hospitals (96.9% versus 72.6%) [19]. In contrast to this finding, our survey did not show a statistically significant difference in awareness between PCCN that worked in tertiary versus general hospitals.

The majority of nurses participating in this survey had over 5 years of experience, yet only 49% in 2006 and 67.9% in 2010 had managed a child with ACS. The low occurrence rate of ACS in children may contribute to the lack of experience with it, since occurrence rates of ACS have been reported as 0.6% to 4.7% in single center mixed pediatric intensive care unit populations [3-5]. It may also reflect the fact that cases of IAH and ACS are going unrecognized. Responses from 27.3% of PCCN in 2006 indicated they never measured IAP, which supports the theory that IAH and ACS cases are likely being missed, as measuring IAP is a key component in the diagnosis of ACS. This percentage has dropped over a 4-year period to 19.1% in 2010 supporting the growing awareness and improved surveillance for ACS.

Surveys reported around 2006 had similar findings of infrequent measurement of IAP among physicians and surgeons working in ICUs. The study of Kimball et al. published in 2006 revealed that 13.2% of members of the Society of Critical Care Medicine (SCCM) had never measured IAP [17]. In a survey among Belgian surgeons, only 41% indicated that they had ever measured IAP [21]. German surgeons and anesthesiologists surveyed in 2009 indicated that 26% were not measuring IAP at the time [22]. A recent survey among Chinese intensivists reported that 30.6% never measure IAP [20].

The purpose of this study was solely to determine awareness of ACS. This survey was not designed to elicit reasons why IAP was not measured. However, a reflection of reasons that IAP is not measured by other healthcare providers may shed light on why some physicians and surgeons incorrectly believe they do not need IAP measurements to diagnose ACS, and a clinical exam is sufficient. Fifty percent of Belgian surgeons relied on clinical exams for diagnosing ACS [21]. For 20% of SCCM members surveyed, the clinical exam was thought to be sufficient to make the diagnosis [17]. Reasons given among intensivists surveyed for not measuring IAP included "think it is futile" - 36.4%, "did not know how to measure" - 27.2%, "did not know how to interpret results" - 33.3, and "did not admit patients with IAH" - 3% [1]. In the survey of Kimball et al., 3.4% of respondents did not use bladder methods to measure IAP because they did not believe there was a clinical correlation. Other healthcare providers used the clinical exam to screen patients and measured IAP when there was clinical suspicion. In a survey of ICUs, 75.9% indicated they measured IAP on some occasion, but only when there was clinical suspicion of IAH or ACS [1]. The method most commonly used to measure IAP, as indicated by virtually all surveys, was the bladder method. This was similar to our findings [1,16-18,20].

Direct experience with measuring IAP is increasing among PCCN. The percentage of nurses having direct experience with its use almost doubled in 2010 as compared to 2006. Simultaneously, there appears to be a drop in the sole use of clinical examination to monitor IAP. This is reassuring, as using clinical examination alone to diagnose IAH and ACS is inaccurate with a sensitivity of about 40% [23].

Though the awareness of ACS and experience in measuring IAP has improved, the understanding of the definition of ACS among PCCN still appears to be unclear with possibly even greater confusion over time. Half of PCCN in our survey currently define ACS as an elevation in IAP associating it with a specific threshold value and not with development of organ dysfunction. The problem understanding what comprises ACS extends to other non-nurse healthcare providers as well. Burke and Latenser's survey of burn physicians indicated that 15% did not include clinical sequelae in their definition of ACS [18]. Only 46.8% of pediatric HCP in a survey understood the development of new organ dysfunction or failure associated with an elevation in IAP constituted the definition of ACS [16]. The threshold used to define IAP was variable with the majority indicating the threshold at which ACS would likely develop to be 15-25 mmHg. Surveys among other healthcare providers working in ICUs had similar findings with critical IAP ranging from 11 to 50 mmHg [17-19,22,24]. The critical IAP at which organ dysfunction develops varies among individuals, and this perhaps plays a role in the lack of clarity regarding ACS definition among PCCN and healthcare providers working in ICUs.

In children, the variation in IAP threshold at which ACS occurs may be even more pronounced based on normal physiology. This is best explained using the concept of APP which is the difference between mean arterial pressure (MAP) and IAP [7,25]. This concept is similar to the concept of cerebral perfusion pressure in patients with intracranial hypertension [2]. Smaller children generally have lower MAP than adults therefore could theoretically be at greater risk of developing ACS at lower IAP than adults. This can be due to the fall in APP to lower levels. Keeping the pathophysiology of ACS in mind while managing patients will assist in making the diagnosis of ACS in situations where ACS occurs at lower IAP thresholds than the classical definition using 20 mmHg in adults. Several pediatric studies have reported the occurrence of ACS at thresholds as low as 12 and 17 mmHg [3-5,26]. Providers must understand that the WSACS definition is useful in adult patients but not directly applicable to pediatric patients. These factors may all contribute to the apparent confusion in the definition demonstrated in this survey. More studies to better understand IAP thresholds or APP thresholds associated with ACS are needed to bring clarity to the definition of ACS as it pertains to children. ACS definitions specific to children are essential. There is a need for focused education on ACS recognition and clinical significance among PCCN because of their vital role as members of the ICU team and "first-responders" at the bedside in the ICU.

## Conclusion

This study indicates that there is a growing awareness of ACS among PCCN. However, there is also significant confusion about the definition of ACS which may delay its recognition by PCCN at the bedside. There is a need for targeted education regarding the recognition and treatment of ACS in nursing curricula, as well as preceptorships in the pediatric critical care setting. These steps may enhance practical training regarding measurement and interpretation of IAP in the care of critically ill children.

## Abbreviations

ACS: abdominal compartment syndrome; IAP: intra-abdominal pressure; IAH: intra-abdominal hypertension; PCCN: pediatric critical care nurses; WSACS: World Society of Abdominal Compartment Syndrome

## Competing interests

The authors declare that they have no competing interests.

## Authors' contributions

JN contributed to the design of this study, participated in distributing and collecting the questionnaires, and wrote portions of the manuscript. MM helped develop the idea and design the study. He wrote portions of the manuscript and played a key role in editing the manuscript. KB helped in the design of the questionnaire and performed the statistical analysis needed for validation of the questionnaire and all the data analysis for the study. JCE conceived the study idea, developed and designed the study, as well as wrote and edited portions of the manuscript. All authors read and approved the final manuscript.

## Authors' information

JN is a nurse practitioner working in the Cardiac Intensive Care Unit at Loma Linda University Children's Hospital. MM is a subspecialist in Pediatric Critical Care Medicine and Director of the Pediatric Critical Care Fellowship at Loma Linda University Children's Hospital. Research interests include abdominal compartment syndrome, brain death in children, and organ donor management. KB is a statistician with a great deal of experience with clinical studies. JCE is a subspecialist in Pediatric Critical Care Medicine and Medical Director of the Cardiac Intensive Care Unit at Loma Linda University Children's Hospital. Research interests include understanding abdominal compartment syndrome and intra-abdominal hypertension in children.

## Supplementary Material

Additional file 1 **Appendix. Abdominal Compartment Syndrome Awareness Questionnaire**. Click here for file
