# Supplementary material for: Pediatric critical care nurses' experience with abdominal compartment syndrome
Source: Ann Intensive Care. 2012 Jul 5;2(Suppl 1):S6. doi: 10.1186/2110-5820-2-S1-S6 (PMC3390293; doi:10.1186/2110-5820-2-S1-S6)
Supplement: Additional file 1 — Appendix. Abdominal Compartment Syndrome Awareness Questionnaire. [file 2110-5820-2-S1-S6-S1.pdf]

Appendix

Abdominal Compartment Syndrome Awareness Questionnaire

Please circle your appropriate response. Completion of this questionnaire is voluntary.

1. What is your profession?
   a. General pediatrician
   b. Pediatric nurse
   c. Pediatric surgeon
   d. Pediatric critical care physician
   e. Other______________________
   (fill in your profession)

2. What type of institution are you in?
   a. Tertiary institution
   b. Community hospital
   c. Private practice
   d. Clinics
   e. Others_____________________
   (fill in type of practice)

3. Where do you practice?
   a. United States of America
   b. Europe
   c. Other _____________________
   (fill in country of practice)

4. Do you work in an intensive care unit?
   a. No
   b. Yes

5. How long have you been in practice?
   a. 0-5 years
   b. >5-10 years
   c. >10 years

6. Have you heard of abdominal compartment syndrome (ACS)?
   a. No
   b. Yes

7. Have you managed/cared for a child (0-18 years) with ACS?
   a. No
   b. Yes

8. Do you measure intra-abdominal pressures (IAP) during your management/care of ACS?
   a. No
   b. Yes
   c. Sometimes

9. What method have you used to measure IAP? (Circle all that apply)
   a. Clinical palpation
   b. Bladder method
   c. Direct intra-peritoneal method
   d. Intra-esophageal/gastric method
   e. Other______________________
   ___________(fill in method used)

10. How high would the intra-abdominal pressures have to be before you call it ACS?
    a. When IAP is 0-10 mmHg
    b. When IAP is 10-15 mmHg
    c. When IAP is 15-25 mmHg
    d. When IAP is >25 mmHg
    e. Multi-system organ failure with evidence of elevated abdominal pressures.
    f. other______________________
    ____
    ____
